# Traumatic Tetanus

**Published:** 1902-02

**Authors:** 


### TRAUMATIC TETANUS.

Seen first August 26, 1901. Injected hypodermically carbolic acid, 95 per cent. strength, one-half drachm; glycerin, one and a half ounces; aquæ destillatæ, three fluidounces. Injections were made at the shoulder region, where but slight swelling followed. Injections heretofore made in the neck were followed by extensive sloughing; much of the sloughing region had to be extirpated with the knife, under which fistulous tracts six to seven inches long were noticed. A 20 per cent. creolin solution was used in washing and injecting the fistulous tracts, after which iodoform 8 parts and tannic acid 1 part, as a dressing, was followed by a rapid healing of the same and recovery from the tetanus.

---

The New Jersey Board of Agriculture have adopted resolutions recommending to the State Legislature the passage of the proposed measure to establish a State Board of Veterinary Medical Examiners.

